# Characterization of food portion size in children from 6 months to 8 years of age: a descriptive analysis

**DOI:** 10.1007/s00394-026-03943-7

**Published:** 2026-03-24

**Authors:** Irene Hernandez-Perez, Joaquin Escribano, Veit Grote, Berthold Koletzko, Mariona Gispert-Llauradó, Mireia Alcázar, Elvira Verduci, Dariusz Gruszfeld, Louise Etienne, Veronica Luque, CHOP Study Group, H Demmelmair, U Handel, I Pawellek, S Schiess, S Verwied-Jorky, M Weber, R Closa-Monasterolo, M Gispert-Llauradó, C Rubio-Torrents, M Zaragoza-Jordana, A Dobrzańska, D Gruszfeld, R Janas, A Wierzbicka, P Socha, A Stolarczyk, J N Van Hees, J Hoyos, J P Langhendries, F Martin, P Poncelet, A Xhonneux, C Agostoni, M Giovannini, S Scaglioni, F Vecchi

**Affiliations:** 1 Paediatrics, Nutrition and Development Research Unit, Universitat Rovira I Virgili, Reus, Spain; 2 Institut de Recerca Biomèdica CatSud, Reus, Spain; 3 German Center for Child and Adolescent Health, site Munich, Department of Paediatrics, LMU - Ludwig Maximilians Universität Munich, Dr. Von, Hauner Children’s Hospital, LMU University Hospitals, 80337 Munich, Germany; 4 Metabolic Diseases Unit, Department of Paediatrics, Vittore Buzzi Children’s Hospital, University of Milan, Milan, Italy; 5 Department of Health Sciences, University of Milan, Milan, Italy; 6 Neonatal Department, Children’s Memorial Health Institute, Warsaw, Poland; 7 Groupe Santé CHC, Bd. Patience Et Beaujonc 2, 4000 Liège, Belgium; 8 Serra Hunter Fellow, Universitat Rovira I Virgili, Tarragona, Spain

**Keywords:** Complementary feeding, Portion size, Infant nutrition, Feeding practices, Childhood nutrition

## Abstract

**Purpose:**

Diet during early infancy, as well as dietary patterns during childhood and parental feeding styles influence children’s eating behaviours and long-term health. While extensive data exist on the timing of complementary feeding introduction and recommended food frequencies during infancy and childhood, data on portion sizes during this period remains limited in Europe, despite their clear relevance for evaluating dietary patterns and supporting evidence-based guidance. This longitudinal study, secondary to the European Childhood Obesity Project (EU CHOP), aims to describe portion sizes consumed by infants and children aged 6 months to 8 years across five European countries.

**Methods:**

Dietary intake was recorded using 3-day food diaries and analysed by trained personnel following standardized procedures at multiple infant and childhood ages (6, 7, 8, 9, 12, 24, 36, 48, 60, 72 and 96 months). Portions sizes were calculated for 33 food groups and expressed as percentiles in the overall sample and stratified by country.

**Results:**

A total of 1018 3-day food records were available at 6 months, with sample size gradually declining over the 11 follow-up time points to 400 records at 8 years. The results revealed a wide variation in portion sizes across food groups, ages and countries.

**Conclusion:**

Food portion sizes vary across food groups, age and countries. These findings provide reference percentiles representing typical portion sizes when foods are consumed. The data can support guidance provided to parents by healthcare professionals (pediatricians, nurses, and dietitian-nutritionists) and assist public health authorities in defining appropriate portion sizes and mitigating risks associated with both overeating and undereating, while respecting children’s hunger and satiety cues. These results also have practical applications in school meal planning and dietary assessment methodologies in research.

**Supplementary Information:**

The online version contains supplementary material available at 10.1007/s00394-026-03943-7.

## Background

Complementary feeding and dietary patterns during early childhood play a critical role in establishing long-term eating habits and influencing later health outcomes[1–3]. Studies have mainly examined the food quality and frequency of consumption during this period [4, 5].

There is a significant amount of information available regarding the timing of introducing complementary feeding, and which foods should be consumed more or less frequently [6–8]. Evidence suggests that eating vegetables, fruits, pulses and nuts 3 or more times per week has been inversely associated with body mass index (BMI) in adolescents and children [9]. One study reporting on the consumption of ultra-processed foods and sweetened beverages, show alarming trends: nearly 70% of children aged 2 to 4 years consume artificial juice and sweet biscuits, a habit associated with adverse growth outcomes, including elevated BMI [10]. However, while much of the existing evidence focuses on diet quality and frequency of consumption, the role of portion sizes in childhood has been studied to a lesser extent, despite their clear relevance for evaluating dietary patterns and supporting evidence-based guidance [11].

Infants are born with an innate ability to regulate food intake, in response to hunger and satiety cues [12]. However, as children grow and become increasingly influenced by external feeding signals, the efficacy of this natural self-regulation tends to decline [13]. Food portion or portion size, generally defined as the amount of food consumed in a single eating occasion [14], has been associated with body weight in toddlers [15].

Highly controlling parental feeding styles, such as food restriction, can contribute to the development of unhealthy eating habits or dysregulated food intake that may persist into adulthood [16, 17]. Conversely, a responsive feeding approach that includes offering age-appropriate food portions and respecting children satiety cues may support an adequate self-regulation [14, 15]. Recent data indicate that many parents —especially first-time parents— have limited knowledge about appropriate portions sizes, which may increase the risk of overfeeding in infants [18]. Caregivers reported to often base their decisions on personal experience or information found on websites [18].

The family environment, including factors such as educational level and socioeconomic status, plays a critical role in shaping a child’s diet [19]. Parents significantly influence their children’s food preferences, as well as dietary quality and quantity [19, 20]. In turn, parents’ food choices for their children are influenced by various factors, including food packaging, advertising and marketing campaigns designed by industry to attract consumers [21]. Moreover, the portion size of many ultra-processed food products marketed to adults has increased over the past several decades, which may contribute to a normalization of larger servings – even for children [22, 23].

Although existing European guidelines mainly focus on which foods should be introduced and recommended frequencies, they do not provide guidance on actual portion sizes per eating occasion [24, 25]. While portion sizes during complementary feeding were documented in a United States paediatric population in 2006 using household measures [26], similar data for infants in European populations are lacking. Most available evidence on portion size in children has focused on analytical associations with energy intake and adiposity but descriptive data across early childhood remain limited. A recent systematic review reported predominantly positive associations between larger portion sizes and indexes of adiposity; however, most included studies were cross-sectional and conducted in school-aged children and adolescents, with scarce information from infancy and the complementary feeding period [27].

Overall, the limited impact and reach of existing guidelines underscore the need for robust descriptive data to better inform the development of future nutritional recommendations and interventions at the European level [28–30].

The age range included in this study, from 6 to 96 months, covers distinct developmental stages characterised by different feeding skills and self-regulation abilities. Considering these developmental variations is essential for interpreting portion size data and informing age-appropriate dietary recommendations. The aim of this study was to describe food portion sizes consumed by infants and children aged 6 months to 8 years across five European countries.

## Methods

### Study design and population

This is a longitudinal analysis secondary to the EU Childhood Obesity Project (EU CHOP). The EU CHOP was formerly a double-blind, randomised clinical trial testing whether reducing protein supply during the first year of life could reduce later obesity risk. The EU CHOP trial was registered at clinicaltrials.gov on June 19th, 2006 (Clinical trial registration number: NCT00338689).

Participants were enrolled during the first 8 weeks of life and were recruited in the neonatal unit of hospitals from 5 European countries: Belgium (Brussels and Liege), Germany (Munich and Nuremberg), Italy (Milano), Poland (Warsaw) and Spain (Tarragona and Reus).

Inclusion criteria for the study were apparently healthy, singleton, term infants born between October 2002 and July 2004. Participants had to reside in the study area and caregivers had to be fluent in the local language. Infants whose mothers ingested drugs during pregnancy that could influence infant growth or mothers with hormonal and/or metabolic diseases were excluded. Formula-fed participants were randomised to receive one of two non-hydrolysed cow’s milk nutritionally complete formulas with different protein content. A group of breastfed infants was also recruited for comparison [31]. Follow-up visits were conducted monthly from birth until 9 months and at 12 and 18 months. Afterwards, annual visits were conducted from 2 to 8 years of age. Data collection was performed between 2002 and 2012. The sample size of the original clinical trial was calculated to detect differences in growth outcomes between the intervention groups (> 500 on each group), as reported previously [31]. All children with available dietary intake data were included in the present secondary analysis. The decreasing sample over time resulted from a combination of factors, including loss to follow-up, missing dietary records, or records not meeting accuracy criteria (detailed below); no participants were excluded for other reasons. More details of the project and results have been previously published [31, 32].

### Dietary intake assessment

Food intake from 6 months to 8 years of life (6, 7, 8, 9, 12, 24, 36, 48, 60, 72, and 96 months) was recorded by parents or caregivers over three consecutive days, including two weekdays and one weekend day. Food records were specifically designed for different age ranges (weaning period, toddlers’ period and ≥ 3 years of life) to improve the data collection accuracy and quality. Dietitians completed standardized training using 1-day food records containing examples from participant diaries. All training entries were centrally reviewed by a responsible dietitian, and any differences were resolved through consensus to harmonize practices.

Parents were provided with a digital scale (Unica 66006; Sohenle, Murrhardt, Germany, with precision of 2 g below 1 kg and 5 g above 1 kg) and detailed instructions on how to weigh and record foods. When weighed was not possible, portion sizes were estimated through food pictures atlas (self-designed by the EU CHOP study nutritionist). Parents were instructed to record both offered and consumed amounts [33, 34]. In addition, all returned 3-day food records were systematically checked by trained nutritionists according to standard operating procedures, with clarification sought from caregivers when necessary. Data entry of food records was performed in a dedicated software (Nutrcalc), which was developed as a part of the EU CHOP project. Procedures were implemented to ensure consistent data entry and scoring across study centers [34]. Food records quality was assessed based on 4 items: Food description accuracy, information about the amount of recipe ingredients, quantity offered and consumed. Each day of the 3-day food records introduced on Nutrcalc received an accuracy score: 0 = absent, 1 = poor, 2 = fairly good, 3 = good. Food records with absent information or poor accuracy were excluded from this analysis. All dietary diaries were screened for implausible portion sizes and were classified as implausible if it lacked consistent periodicity in consumption and/or exceeded three times the age specific 90th percentile for the corresponding food group. For example, a 610 g vegetable portion reported for a 7-month-old infant was considered as implausible and excluded, whereas a 400 ml milk portion at 2 years was retained when similarly high—but still physiologically plausible—intakes were observed (e.g., 350 and 380 ml).

Food items were classified primarily according to the standard food groups and subgroups of the German BLS II.3 food composition database [33]. A SOP protocol was developed to ensure harmonized classification across countries. Local food items not included in the BLS database were systematically assigned to existing groups when possible, or to newly adapted groups following the Food Classification SOP, based on nutritional composition and ingredient information from manufacturers, national food composition tables, and product labels. Mixed dishes were disaggregated into individual ingredients for analysis. When families provided records with each ingredient specified, these data were used directly. Otherwise, standardized recipes from each study centre were applied to estimate the composition of the dish.

### Food portion calculation

Food items were classified in 33 food groups (see Supplementary Table 1, Online Resource 1). When multiple items from the same food group were consumed in the same meal (e.g., carrots and tomatoes) they were combined and treated as a single portion of that food group. Their weights were summed (rather than averaged) to reflect total food group intake per meal (values expressed in grams). For each participant, the mean portion size per food group was calculated across all meals in which that food group was consumed, representing the portion size when actually consumed, rather than overall intake. If a food group was consumed in multiple meals within the same day, each eating occasion was treated separately. A mean portion size was derived by averaging the portion sizes across all meal occasions when the food group was consumed.

For example, if a child consumed bread at breakfast on Day 1(30 g), lunch on Day 1(25 g), breakfast on Day 2 (35 g) and not consumed during Day 3, the mean portion size across all eating occasions was (30 + 25 + 35 g) / 3 = 30 g. A Supplementary figure 2 illustrates the calculation procedure (see Online Resource 2).

### Other variables

Sociodemographic information was collected at enrolment (< 14 weeks of age) during an interview.

### Anthropometry

All anthropometric measurements were performed by trained dietitians, nutritionists or study nurses, who received instructions on standardized procedures and completed 4 h of training sessions four times throughout the study. The same equipment, periodically calibrated, was used in all the centres: SECA 702 for weight and SECA 242 stadiometer for height was use at 8 years. Children were classified as overweight or obesity category if their BMI was > 1 SD according to the World Health Organization criteria [34].

### Statistical analyses

The characteristics of the study sample are described as mean and Standard Deviation (SD) (quantitative variables) and frequencies and percentages (categorical variables). For the comparison of baseline characteristics between the initial and final analysed populations, Chi-square test was used for categorical variables, and the Mann–Whitney U test was applied for continuous variables that were not normally distributed (Kolmogorov–Smirnov test, p < 0.05).

Portion sizes by age (6, 7, 8, 9, 12, 24, 36, 48, 60, 72, and 96 months) are presented in percentile tables (expressed in grams). Percentiles of food group intakes by age were calculated empirically whenever data were available for that food group and age. No imputation was performed for missing dietary data.

To assess differences between countries, the normality of dietary intake for each food group was evaluated using the Kolmogorov–Smirnov test. As most distributions were not normally distributed, non-parametric statistical methods were applied: the Kruskal–Wallis test was used for overall comparisons, and pairwise comparisons were performed with Bonferroni adjustment.

A sensitivity analysis was conducted to describe food portion sizes across infancy and childhood in children without overweight or obesity at age of 8 years, aiming to assess whether the portion sizes presented in this article might be excessive or potentially unhealthy.

Data management and statistical analyses were carried out with the software packages SPSS Statistics (Version 29.0.1.0) and RStudio (Version 4.5.2) [35].

## Ethics

Ethical approval was obtained; see Declarations (section “Ethics approval and consent to participate”) for details.

## Results

### Description of the study sample

A total of 1679 infants were recruited (1090 infants in the intervention arms). At 6 months, dietary intake was available for 1018 children, with the number decreasing over time to 400 children at 8 years of age. Table 1 shows the baseline characteristics of the population at the beginning and the end of the descriptive analysis (6 and 96 months). Supplementary Fig. 1 shows the participants included in the descriptive analysis at each time point by country (see Online Resource 3).

**Table 1:** Baseline characteristics for all participants providing dietary intake data at 6 and 96 months

		6 months	8 years	
		n (%)	n (%)	*p* value
Country	*Total*	1018 (100)	400 (100)	< 0.001*
*Germany*		168 (16.5)	60 (15)	
*Belgium*		125(12.3)	30 (7.5)	
*Italy*		273 (26.8)	76 (19)	
*Poland*		190 (18.7)	83 (20.8)	
*Spain*		262 (25.7)	151 (37.8)	
Gender	*Female*	528 (51.8)	207 (51.7)	0.969
Formula	*Low protein*	387 (38)	137 (34.3)	0.004*
*High protein*		372 (36.5)	126 (31.5)	
*Breastfed*		259 (25.4)	137 (43.3)	
Birth order	*First child*	589 (58)	229 (57.3)	0.328
*Second child*		327 (32.2)	139 (34.8)	
> *Second child*		100 (9.8)	32 (8)	
Spontaneous delivery		702 (69.2)	264 (66)	0.240
Mother’s education	*Low*	245 (24.1)	60 (15)	< 0.001*
	*Middle*	520 (51.2)	201 (50.2)	
	*High*	251 (24.7)	138 (34.5)	
Father’s education	*Low*	271 (26.9)	82 (20.5)	0.009*
	*Middle*	501 (49.9)	199 (49.8)	
	*High*	233 (23.2)	118 (29.5)	
Single mother		51 (5)	12 (3)	0.097
Mother smoked during pregnancy		350 (34.5)	115 (28.7)	0.041*
BMI mother before pregnancy				0.983
< *20*		117 (12)	68 (17.4)	
*20–25*		521 (53.3)	208 (52)	
*25–30*		200 (20.5)	83 (20.8)	
> *30*		79 (8)	31 (7.8)	
		mean (SD)	mean (SD)	*p* value
Weight (kg)		3.30 (0.35)	3.29 (0.33)	0.444
Height (cm)		50.65 (2.60)	50.73 (2.64)	0.235
Weight-for-length (z-score)		−0.70 (1.47)	−0.77 (1.50)	0.345
Weight-for-age (z-score)		0.00 (0.74)	−0.01 (0.71)	0.616
Head circumference (cm)		34.22 (1.35)	34.20 (1.37)	0.446
Born week		39.82 (1.21)	39.85 (1.21)	0.610

Pearson chi-square tests were used for statistical comparison of categorical data, and a U- de Mann Whitney was used for non-normally distributed continuous data.

No significant differences were observed in sex distribution, infant anthropometric measures, gestational age at birth, single mother status, type of delivery, and maternal BMI before pregnancy between the groups analysed (6 months; 8 years). However, significant differences were identified for country, type of formula, parental educational level and mother smoking during pregnancy. Parents from children with food records at 8 years of age had a higher educational level, and mothers were more likely to be non-smokers during pregnancy compared with those who were originally recruited. At 8 years of age, children of mother who breastfed were more likely to have completed food record (Table 1).

At 8 years of age, anthropometric measurements were obtained for 582 participants. Of these, 170 (29.2%) were classified in the overweight or obesity group, while 412 were classified in the normal weight group, including 7 children categorized as underweight.

### Food portion size description

Of a total of 44,353 food record days across the study period, 3,180 (7.2%) were excluded due to poor accuracy. Food portion sizes (in grams) from 6 to 96 months of age are presented as percentiles (p10, p25, p50, p75, p90) across different ages are shown in Tables 2 (from 6 to 9 months of age), 3 (from 12 to 36 months of age) and 4 (from 48 to 96 months of age), as complementary feeding is generally recommended starting around 6 months currently. Food items included in each food group are listed in Supplementary Table 1 (see Online Resource 1).

**Table 2:** Food portion size (grams) of foods commonly consumed by infants between 6 and 9 months of age. n (%) defines the number of children consuming the food group

Food groups (grams)	Age (months)																							
	6						7						8						9					
	n = 1018						n = 949						n = 911						n = 898					
*Percentile*	n (%)	10	25	50	75	90	n (%)	10	25	50	75	90	n (%)	10	25	50	75	90	n (%)	10	25	50	75	90
Fruits	407 (40.0)	42	71	118	163	214	488 (51.4)	46	71	115	170	220	489 (53.7)	40	71	110	165	213	530 (59.0)	46	67	100	250	209
Processed fruits products	469 (46.1)	40	65	87	130	190	523 (55.1)	46	75	97	130	190	537 (59.0)	53	80	100	134	200	531 (59.1)	60	80	105	140	200
Vegetables	401 (39.4)	8	14	45	85	130	602 (63.4)	10	20	55	102	139	614 (67.4)	11	22	60	103	135	640 (71.3)	11	27	58	101	145
Leafy vegetables	231 (22.7)	< 1	1	2	8	21	316 (33.3)	1	2	4	11	45	341 (37.4)	1	2	5	15	50	364 (40.5)	1	2	6	15	57
Soups and vegetable dishes	205 (20.1)	36	62	110	151	190	207 (21.8)	37	69	113	165	190	222 (24.4)	40	69	101	175	190	214 (23.8)	47	70	90	190	215
Ready-to-eat infant foods	422 (41.5)	6	27	69	130	190	538 (56.7)	29	52	99	169	192	574 (63.0)	39	66	117	184	210	538 (59.9)	40	69	131	190	220
Cow milk and regular yoghurt	875 (86.0)	123	153	181	208	230	828 (87.2)	119	149	180	210	235	816 (89.6)	116	149	180	214	237	826 (92.0)	115	147	180	214	240
Flavored milks	16 (1.6)	49	62	100	120	133	65 (6.8)	43	50	75	122	125	101 (11.1)	45	53	100	125	139	183 (20.4)	50	60	100	125	137
Processed milk products	79 (7.8)	10	21	40	70	100	185 (19.5)	15	27	50	80	116	210 (23.1)	15	27	50	80	116	236 (26.3)	11	21	50	80	104
Cheese	227 (22.3)	3	4	5	11	23	264 (27.8)	4	7	14	22	50	290 (31.8)	5	9	17	30	80	334 (37.2)	5	10	17	30	80
Grains	794 (78.0)	5	8	14	22	38	836 (88.1)	6	11	16	24	44	840 (92.2)	7	11	16	26	50	860 (95.8)	8	12	19	29	53
Potatoes	369 (36.2)	5	9	29	60	91	562 (59.2)	6	14	43	72	100	574 (63.0)	7	17	45	76	102	600 (66.8)	7	19	47	78	106
Pulses	2 (0.2)	1	1	6	N/R	N/R	5 (0.5)	5	5	16	26	N/R	14 (1.5)	1	5	13	35	93	24 (2.7)	2	5	19	54	138

Food groups (grams)	Age (months)																							
	6						7						8						9					
	n = 1018						n = 949						n = 911						n = 898					
Percentile	n (%)	10	25	50	75	90	n (%)	10	25	50	75	90	n (%)	10	25	50	75	90	n (%)	10	25	50	75	90
Red meat	46 (4.5)	7	15	20	38	65	156 (16.4)	12	19	30	42	61	222 (24.4)	10	20	30	50	75	289 (32.2)	13	20	31	50	74
White meat	88 (8.6)	8	14	24	44	56	286 (30.1)	10	20	31	50	72	312 (34.3)	14	20	34	51	74	317 (35.3)	12	20	32	50	70
Processed meat	16 (1.6)	ND	1	8	19	47	50 (5.3)	1	2	12	38	46	89 (9.8)	1	10	25	38	75	157 (17.5)	1	6	20	35	60
Eggs	9 (0.9)	1	2	5	24	N/R	65 (6.8)	2	4	10	12	29	84 (9.2)	2	8	10	19	29	132 (14.7)	2	6	14	22	36
Fatty fish	-	N/R	N/R	N/R	N/R	N/R	3 (0.3)	2	2	27	N/R	N/R	4 (0.4)	25	31	67	96	N/R	10 (1.1)	2	8	25	57	100
Lean fish and seafood	7 (0.7)	13	17	19	40	N/R	31 (3.3)	13	16	30	55	94	59 (6.5)	16	23	45	68	100	127 (14.2)	18	30	50	70	93
Oils	345 (33.9)	2	3	5	5	9	496 (52.3)	2	4	5	6	9	511 (56.1)	2	4	5	6	10	533 (59.4)	2	4	5	6	10
Other sauces (not oil based)	-	N/R	N/R	N/R	N/R	N/R	11 (1.2)	< 1	< 1	1	8	9	15 (1.7)	N/R	1	1	4	16	35 (3.9)	< 1	< 1	1	3	11
Saturated spreads	62 (6.1)	< 1	1	3	5	8	120 (12.6)	< 1	1	3	5	6	144 (15.8)	1	1	3	5	7	180 (20.0)	0	1	3	5	7
Added sugar	65 (6.4)	< 1	2	3	5	7	76 (8.0)	1	2	4	7	10	111 (12.2)	< 1	3	5	10	16	148 (16.5)	1	3	5	9	13
Cakes, biscuits and pastries	69 (6.8)	5	9	14	21	28	111 (11.7)	5	6	14	20	25	181 (19.1)	5	7	10	16	29	227 (25.3)	6	8	13	20	30
Confectionary	–	N/R	N/R	N/R	N/R	N/R	-	N/R	N/R	N/R	N/R	N/R	9 (1.0)	2	6	10	64	N/R	17 (2.0)	2	5	12	20	30
Savory snacks	–	N/R	N/R	N/R	N/R	N/R	8 (0.8)	< 1	1	5	12	N/R	13 (1.4)	1	5	8	20	77	14 (1.6)	< 1	1	3	7	45
Beverages	566 (55.0)	16	30	51	75	110	558 (58.7)	17	30	50	87	120	530 (58.2)	18	31	53	87	125	539 (60.0)	18	33	58	100	135

n: number of children reporting dietary intake data at each age; n (%)*: number (percentage) of children reporting consumption of the specific food item at each age; consumption of processed cereal products, processed potatoes, processed fish, soft drinks and nuts and seeds were not reported in this age period; N/R Not reported in the specific age or Not Reported by SPSS Statistics due to the small number of observations or low data variability (percentiles coincide with other values or cannot be reliably estimated).

Between 6 and 12 months of age, 40–59% of infants consumed fresh fruits and 46–59% consumed processed fruit products during the 3-day recording period. At this age, fruit portion sizes ranged from 40 to 220 g when considering both fresh and processed products. The relatively largest portions of “Fruits” were consumed between 6 and 9 months of age, while the largest portions of “Processed fruit products” were consumed between 12 and 36 months, however, “Processed fruit products” were consumed by a smaller percentage of participants than “Fruits” (e.g., at 18 months, 84.3% vs. 28.2%, respectively).(Table 3).

**Table 3:** Food portion size (grams) of foods commonly consumed by toddlers and young children from 1 to 3 years of age. n (%) defines the number of children consuming the food group

Food groups (grams)	Age (months)																							
	12						18						24						36					
	n = 886						n = 740						n = 754						n = 534					
*Percentile*	n (%)	10	25	50	75	90	n (%)	10	25	50	75	90	n (%)	10	25	50	75	90	n (%)	10	25	50	75	90
Fruits	626 (70.6)	38	58	95	140	203	624 (84.3)	38	58	86	123	173	640 (84.9)	35	55	81	110	141	461(86.3)	41	65	88	119	140
Processed fruits products	444 (50.1)	70	80	100	152	209	209 (28.2)	74	80	100	170	250	96 (12.7)	77	85	111	190	250	36 (6.7)	80	80	120	130	250
Vegetables	728 (82.2)	12	27	54	94	134	704 (95.1)	14	27	47	77	115	739 (97.0)	13	24	40	64	98	522 (97.8)	14	25	42	63	88
Leafy vegetables	405 (45.7)	1	2	6	15	50	396 (53.5)	N/R	1	5	15	40	445 (59.0)	N/R	1	5	16	36	349 (65.4)	1	1	6	17	36
Soups and vegetable dishes	145 (16.4)	41	74	80	188	222	48 (6.5)	20	56	80	190	253	26 (3.5)	47	75	131	233	258	14 (2.6)	37	80	125	200	261
Ready-to-eat infant foods	474 (53.5)	57	80	146	210	250	176 (23.8)	52	80	163	234	150	99 (13.1)	60	115	190	250	250	20 (3.8)	61	80	160	250	250
Cow milk and regular yogourt	845 (95.4)	107	145	180	213	240	705 (95.3)	94	138	179	215	250	717 (95.0)	78	125	171	215	250	509 (95.3)	78	121	162	206	250
Flavored milks	329 (37.1)	45	55	100	125	138	441 (59.6)	41	70	105	125	150	471 (62.5)	41	70	105	125	150	314 (58.8)	50	75	110	125	168
Milk products processed	265 (29.9)	6	15	50	97	120	266 (36.0)	4	9	16	30	73	279 (37.0)	3	7	14	32	60	243 (45.5)	5	10	19	45	75
Cheese	505 (57.0)	5	10	20	50	100	580 (78.4)	5	10	20	43	100	623 (82.6)	5	10	20	43	90	469 (87.8)	5	10	18	33	69
Grains	873 (98.5)	12	17	23	34	53	736 (99.5)	16	21	29	39	54	752 (99.7)	18	24	33	43	57	533 (99.8)	23	30	39	51	63
Processed cereal products	8 (0.9)	2	21	43	65	N/R	25 (3.4)	26	38	50	70	90	55 (7.3)	28	40	55	80	124	38 (7.1)	29	50	65	77	102
Potatoes	662 (74.7)	10	25	53	85	113	599 (81.0)	18	37	61	92	123	606 (80.4)	20	36	58	92	123	419 (78.5)	25	41	60	99	134
Processed potatoes	10 (1.1)	10	20	50	100	156	37 (5.0)	26	50	60	100	156	49 (6.5)	26	50	60	100	155	51 (9.6)	38	50	55	82	100
Pulses	74 (8.4)	6	10	32	62	100	101 (13.8)	8	16	50	97	139	102 (13.8)	5	18	32	100	150	62 (11.8)	11	28	59	99	162
Nuts and seeds	23 (2.7)	< 1	2	3	10	33	61 (8.3)	N/R	N/R	1	5	8	101 (13.4)	N/R	N/R	2	7	15	80 (15.0)	N/R	N/R	3	10	20

Food groups (grams)	Age (months)																							
	12						18						24						36					
	n = 886						n = 740						n = 754						n = 534					
*Percentile*	n (%)	10	25	50	75	90	n (%)	10	25	50	75	90	n (%)	10	25	50	75	90	n (%)	10	25	50	75	90
Red meat	386 (43.6)	12	20	35	53	80	508 (68.6)	14	25	40	60	91	598 (79.3)	13	24	40	62	97	439 (82.2)	14	27	41	60	84
White meat	397 (44.8)	15	24	39	60	94	412 (55.7)	21	30	50	70	100	429 (56.9)	20	33	50	78	100	273 (51.1)	23	40	58	77	110
Processed meat	405 (45.7)	4	15	26	40	60	520 (70.3)	8	20	30	49	83	610 (80.9)	12	20	32	50	80	465 (87.1)	13	20	31	53	85
Eggs	278 (31.4)	3	8	19	49	60	446 (60.3)	3	8	25	51	60	495 (65.7)	4	10	27	50	60	400 (74.9)	4	8	23	42	60
Fatty fish	34 (3.8)	10	22	41	71	95	65 (8.8)	12	30	40	71	126	85 (11.3)	7	17	36	66	100	98 (18.4)	5	15	35	60	99
Lean fish and seafood	252 (28.4)	21	35	51	80	109	250 (33.8)	27	45	61	100	129	250 (33.2)	24	45	61	100	129	187 (35.0)	25	44	70	100	126
Processed fish	4 (0.5)	20	21	43	60	N/R	33 (4.5)	29	30	60	73	87	66 (8.8)	17	30	54	74	93	68 (12.7)	7	41	60	90	112
Oils	596 (67.3)	3	4	5	7	10	625 (84.5)	2	4	5	8	10	670 (88.9)	2	4	5	8	10	493 (92.3)	2	4	6	8	11
Other sauces (not oil based)	69 (7.8)	1	1	4	7	11	132 (17.8)	1	2	4	8	12	200 (26.5)	1	2	5	10	15	197 (36.9)	1	3	7	11	20
Saturated spreads	346 (39.1)	1	2	3	5	8	481 (65.0)	1	2	4	5	8	562 (74.5)	1	2	3	6	8	420 (78.7)	2	3	5	7	9
Added sugar	250 (28.2)	1	3	5	10	15	410 (55.4)	< 1	3	5	9	13	513 (68.0)	1	3	5	10	14	411 (77.0)	3	5	8	11	15
Cakes, biscuits and pastries	394 (44.5)	6	9	14	24	35	487 (65.8)	8	12	19	30	43	545 (72.3)	11	16	25	40	54	436 (81.8)	15	21	29	40	50
Confectionary	53 (2.6)	2	5	10	20	28	164 (22.2)	5	8	12	20	31	299 (39.7)	5	9	13	21	32	303 (56.7)	4	8	15	22	31
Savory snacks	36 (4.1)	1	1	6	13	38	93 (12.6)	1	5	20	35	66	112 (14.9)	1	4	17	36	70	126 (23.6)	1	5	25	50	99
Beverages	514 (58.0)	20	39	70	110	150	489 (66.1)	21	58	103	147	200	572 (76.3)	32	75	120	180	200	417 (78.1)	48	100	147	200	200
Soft drinks	–	N/R	N/R	N/R	N/R	N/R	13 (1.8)	20	45	120	225	251	46 (6.1)	30	65	100	148	200	80 (15.0)	41	93	125	200	232

n: number of children reporting dietary intake data at each age; n (%)*: number(percentage) of children reporting consumption of the specific food item at each age;; N/R Not reported in the specific age or Not Reported by SPSS Statistics due to the small number of observations or low data variability (percentiles coincide with other values or cannot be reliably estimated).

At 6 months of age, only 39% of children reported consuming vegetables. However, between 7 and 12 months, 60–82% of children consumed vegetables. From 12 months onwards, over 90% of participants reported vegetable consumption. Portion sizes of vegetables were generally smaller than those of fruits, approximately half the size (median portion sizes ranged between 40 and 60 g). The portion and frequency of leafy greens were lower compared to other vegetables.

Cow’s milk and regular yoghurt were the milk and dairy products more frequently reported (more than 85% of the diaries) at all ages. Median portion sizes were ≈ 180 g from 6 to 18 months and 153 to 166 from 3 to 8 years.

While the portions sizes of grains, processed grains and potatoes exhibited a slight progressive increase over time, the portions of meat, fish and egg remained quite similar throughout childhood. (Table 4).

**Table 4:** Food portion size (grams) of foods commonly consumed by children from 4 to 8 years of age. n (%) defines the number of children consuming the food group

Food groups (grams)	Age (months)	48					60						72						96					
		n = 504					n = 469						n = 447						n = 400					
*Percentile*	n (%)	10	25	50	75	90	n (%)	10	25	50	75	90	n (%)	10	25	50	75	90	n (%)	10	25	50	75	90
Fruits	449 (89.1)	48	73	95	125	148	393 (87.9)	58	80	103	128	150	434 (92.5)	50	77	104	135	158	349 (87.3)	53	78	106	131	150
Processed fruits products	18 (3.6)	74	80	100	130	250	8 (1.8)	30	83	90	100	N/R	9 (1.9)	90	90	95	114	N/R	9 (2.3)	90	90	100	225	N/R
Vegetables	497 (98.6)	18	30	47	65	90	433 (96.9)	17	29	44	65	90	455 (97.0)	21	32	47	68	98	394 (98.5)	23	36	54	83	115
Leafy vegetables	336 (66.7)	1	1	8	25	51	283 (63.3)	1	2	10	30	53	322 (68.7)	1	2	12	34	65	276 (69.0)	1	2	11	35	57
Cow milk and regular yogourt	473 (93.9)	79	120	164	200	249	427 (95.5)	81	120	163	200	248	436 (92.9)	75	112	153	194	230	384 (96.0)	75	125	166	200	250
Flavored milks	302 (59.9)	44	67	110	132	175	276 (61.7)	50	90	120	127	176	292 (62.3)	18	75	110	125	168	234 (58.5)	19	85	125	135	171
Milk products processed	248 (49.2)	5	10	20	50	80	224 (50.1)	5	10	25	51	90	245 (52.2)	7	11	21	50	78	194 (48.5)	10	14	30	60	83
Cheese	442 (87.7)	6	12	19	30	71	397 (88.8)	6	11	20	34	70	423 (90.2)	7	13	20	35	69	350 (87.5)	9	13	22	40	62
Grains	502 (99.6)	28	34	45	58	74	447 (100)	30	38	48	63	79	469 (100)	31	38	51	70	91	399 (99.8)	37	47	65	90	125
Processed cereal products	43 (8.5)	27	37	51	85	100	35 (7.8)	33	50	70	100	127	48 (10.2)	29	52	84	100	131	27 (6.8)	36	70	100	140	155
Potatoes	396 (78.6)	25	44	67	100	135	344 (77.0)	28	46	70	99	135	369 (78.7)	32	50	75	102	146	320 (80.0)	40	65	92	128	170
Processed potaoes	35 (6.9)	39	50	75	100	108	29 (6.5)	50	50	60	100	130	40 (8.5)	50	50	80	100	118	33 (8.3)	50	50	70	100	153
Pulses	70 (13.9)	8	26	63	116	190	73 (16.3)	12	29	63	100	149	62 (13.2)	14	30	61	115	177	60 (15.0)	25	42	88	131	200
Nuts and seeds	115 (22.8)	< 1	< 1	3	10	20	106 (23.7)	< 1	< 1	2	10	23	104 (22.2)	N/R	N/R	4	12	30	78 (20.0)	< 1	1	10	24	41

Food groups (grams)	Age (months)	48					60						72						69					
		n = 504					n = 447						n = 469						n = 400					
*Percentile*	n (%)	10	25	50	75	90	n (%)	10	25	50	75	90	n (%)	10	25	50	75	90	n (%)	10	25	50	75	90
Red meat	436 (86.5)	18	30	47	67	94	378 (84.6)	20	31	50	75	111	406 (86.6)	20	33	52	80	112	341 (85.3)	22	39	60	84	111
White meat	294 (58.3)	27	41	60	80	115	273 (61.1)	28	42	60	86	114	305 (65.0)	34	50	70	90	120	262 (65.5)	40	60	81	110	135
Processed meat	446 (88.5)	14	21	31	50	76	404 (90.4)	13	21	34	51	88	416 (88.7)	15	21	34	50	78	365 (91.5)	16	23	35	50	75
Eggs	386 (76.6)	4	8	20	37	60	336 (75.2)	5	9	20	38	60	366 (78.0)	5	10	21	37	60	336 (84.0)	5	10	27	45	60
Fatty fish	94 (18.7)	8	16	30	71	100	105 (23.5)	9	19	30	67	100	106 (22.6)	10	20	30	55	100	97 (24.3)	9	19	40	56	100
Lean fish and seafood	181 (35.9)	22	42	70	100	148	155 (34.7)	26	40	66	100	134	160 (34.3)	25	49	68	100	140	141 (35.3)	30	50	76	105	160
Processed fish	69 (13.7)	3	28	60	90	111	79 (17.7)	15	50	75	92	120	84 (17.9)	30	50	69	92	134	56 (14.0)	7	37	78	104	127
Oils	469 (93.1)	3	5	7	10	12	419 (93.7)	4	6	8	10	13	449 (95.7)	4	6	8	11	14	385 (96.3)	4	7	9	12	16
Other sauces (not oil based)	193 (38.3)	1	4	6	12	20	157 (35.1)	2	4	9	15	24	201 (42.9)	2	5	9	14	20	191 (47.8)	2	5	10	15	20
Saturated spreads	382 (75.8)	2	3	5	8	11	318 (71.1)	2	4	6	8	10	350 (74.6)	3	4	6	8	12	296 (74.0)	3	4	7	9	12
Added sugar	410 (81.3)	3	5	9	12	20	388 (86.8)	3	5	9	13	19	413 (88.1)	4	5	9	13	20	359 (89.8)	4	6	10	13	19
Cakes, biscuits and pastries	424 (84.1)	18	24	30	42	54	400 (89.5)	20	27	36	48	60	413 (88.1)	20	28	37	50	63	334 (83.5)	21	30	40	54	70
Confectionary	314 (62.3)	5	10	15	22	33	292 (65.3)	5	9	15	24	37	288 (61.4)	5	10	15	24	41	268 (67.0)	7	10	17	27	43
Savory snacks	151 (30.0)	1	14	30	50	100	140 (31.3)	1	11	30	63	150	174 (37.1)	1	15	30	65	100	117 (29.3)	1	15	25	50	90
Beverages	392 (77.8)	59	107	150	200	200	336 (75.2)	40	101	156	200	202	354 (75.5)	32	105	163	200	210	294 (73.3)	37	119	188	200	235
Soft drinks	88 (17.5)	50	100	150	200	203	126 (28.2)	75	106	173	200	250	138 (29.4)	100	150	200	200	300	138 (34.5)	100	143	200	231	330

n: number of children reporting dietary intake data at each age; n(%)*: number(percentage) of children reporting consumption of the specific food item at each age; consumption of soups and vegetables dishes and ready-to-eat infant foods were not reported in this age period; N/R Not reported in the specific age or Not Reported by SPSS Statistics due to the small number of observations or low data variability (percentiles coincide with other values or cannot be reliably estimated).

### Differences between countries

Descriptive tables for each country are presented in Supplementary Table 2 (see Online Resource 4), as well as the table showing p-values for pairwise comparisons between countries (see Online Resource 5). Overall, countries showed variation in portion sizes across different food groups.

Fruit consumption shows significant differences specially during the 24 first months; up to 18 months of age, fruit portion sizes were larger in Spain and Belgium compared with the other countries; thereafter, values progressively converged across countries. The same pattern was observed for vegetables, with Spain generally reporting the largest portions, followed by Belgium.

A different pattern is observed for grains; during the 12 first months of life, Germany presented the largest portions, whereas by 8 years of age Italy showed a marked increase and the highest portion sizes. Pulses consumption was low at all ages; however, Spain showed the highest proportion of consumers, and portion sizes differed significantly between Spain and Italy.

For both red and white meat, Spain and Italy consistently reported the largest portions. Similarly, lean fish portions were higher in Spain across time points. For example, at 48 months of age, statistically significant differences were found between Spain vs Poland, Italy, and Belgium, but not with Germany. By 8 years of age, these differences were no longer observed.

Added sugar intake increased over time in all countries, both in portion size and in the proportion of consumers, although few statistically significant associations were identified (e.g., at 24 months Belgium showed higher portions than the other countries). A comparable increasing trend was also observed for cakes and biscuits. Regarding ready-to-eat infant foods (consumption up to 18 months), Spain generally presented the largest portions, whereas Italy showed the smallest, with significant differences between Italy and the other countries across time points.

Finally, beverages consumption increased progressively across the analysed time points, with portion sizes approximately tripling from the beginning to the end of the study period.

### Sensitivity analysis

Descriptive comparison of portion sizes indicated that children with healthy weight consumed amounts similar or identical to those of the overall population. For example, at 36 months, the 10th, 25th, 50th, 75th, and 90th percentiles for grain consumption were 23, 30, 39, 51, and 63 g in the overall sample, compared with 23, 29, 38, 49, and 59 g in the healthy-weight subsample. Similar patterns were observed across all food groups and timepoints, with differences generally amounting to only a few grams upper or down. Descriptive results from this sub-analysis are presented in Supplementary Table 3 (see Online Resource 6).

## Discussion

This study is, to our knowledge, the first to provide comprehensive descriptive data on food portion sizes consumed by infants, toddlers and children across a multicentre European cohort.

In general, while portion sizes in most food groups increase with age, fruits and vegetables remain similar across ages. Vegetable portion sizes suggest that vegetables may often be consumed as ingredients in mixed dishes or side servings rather than as a main dish. However, the precise context of consumption (standalone, part of a recipe, or side dish) has not been explored deeply. We observed a high consumption of processed fruit products compared to natural fruit between 6–12 months of life, which might be due to the consumption of infant jars. This may be concerning due to the possibility of having added sugars and some loss of the fruit’s natural flavour [36]. This group include a wide variety of products, ranging from fruit-only jars to fruit combined with biscuits, which allow us to analyse portion sizes but does not permit a detailed assessment on nutritional quality in this case. This early exposure to processed foods could promote a preference for sweet tastes and negatively influence the development of healthy eating habits [37, 38].

The observed decrease in median portion sizes of cow’s milk and regular yogurt with age may be explained by dietary habits progression during the weaning period: at 6 months, these dairy products are consumed in larger amounts as primary sources of nutrition, whereas from that age onward, complementary foods are gradually introduced and combined with other food groups, leading to smaller individual portions of dairies. Similar patterns have been observed in studies analysing breast milk intake [39].

Beyond portion sizes description, our data also provides information on the prevalence of consumption of different foods, both factors revealing a transition in children’s dietary habits, with a progressive shift from natural foods toward processed and sugar-sweetened products [36].We observed a sustained increase in the portion sizes and the proportion of children consuming flavoured dairy products, biscuits, and sweetened beverages from an early age reflects premature exposure to added sugars, contrary to the recommendations from the World Health Organization and the European Society for Paediatric Gastroenterology and Nutrition, which advise avoiding free sugars during the first two years of life and limiting them thereafter [24, 40, 41]. The low prevalence of fish and pulse consumption over the three-day recording period may reflect both genuinely low intake and the naturally lower frequency of these foods within a typical week. It is likely that some children consumed fish or pulses outside the recoded days, and thus this was not captured. In contrast, processed or red meats, despite recommendations for minimal consumption, were frequently reported.

When comparing our findings with those from Fox et al. [26], notable differences in portion sizes were observed during the first two years of life between the European sample in our study and the American toddlers. During the early stage of complementary feeding (6–11 months), average portion sizes among American toddlers fell at the lower range observed in our European data for items such as fruits, yoghurts, vegetable-based soups and dishes. Conversely, an opposite trend was observed for cereals (pasta, rice, pizza), juices, sugary drinks, dairy desserts, eggs, and processed meats, with intakes among Americans exceeding the 50th percentile. Although our results are expressed as percentiles and those of Fox et al. [26] are presented as means ± standard deviations, the contrasting trends reflect meaningful differences in dietary practices between the two populations. A special strength of the data published by Fox et al. [26] was the use of household measures to quantify food portions. This approach offers a practical framework to translate dietary recommendations into a format easily understood by caregivers and the general population. On the other hand, our approach to show common portions as percentiles, offers a greater understanding —and potentially, acceptability— of a wide range of normality.

It is important to emphasize that, during the introduction of complementary feeding, one of the primary goals is promoting the exposure to a broad variety of tastes and textures, rather than achieving specific intake amounts. Early dietary diversity has been positively associated with improved food acceptance and the establishment of healthier eating habits later in life [42–44]. Since breastfeeding (or infant formulas) remains the primary source of nutrients during this period, complementary foods mainly serve sensory and developmental purposes. This early exposure plays a key role in shaping dietary patterns throughout infancy, which have been linked to cardiometabolic outcomes in later childhood [45]. In line with this education strategy, practices such as pressuring children to finish their plates or adhering to overly restrictive meal plans should be discouraged.

These approaches can interfere with a child’s ability to recognise hunger and satiety cues, potentially leading to overeating or inadequate food intake [46]. Parent’s beliefs about how much their children should eat have been shown to be more strongly associated to children’s BMI more than children’s own portion sizes preferences, highlighting the importance on parental guidance [47]. Interventions that educate parents about responsive feeding practices have shown positive results, including lower body mass index (BMI) scores, reduced use of pressure to eat or food as a reward, and a lower incidence of emotional eating in children [48]. Supporting the education programs with a broad range of food portion sizes (e.g., 10th to 90th percentile for the general population) considered adequate may be a valuable tool. Importantly, these reference values are not intended to prescribe a fixed amount that children should eat, but rather to illustrate the substantial variability in portion sizes across individuals and ages, emphasizing that a wide range of intakes can fall within an adequate and healthy pattern.

In addition to guiding families, these descriptive data have practical applications in institutional settings. For example, these references can inform the planning, preparation, and serving of school menus in cafeterias, where children spend a considerable proportion of their day. They can also support research efforts by enabling proxy estimates of energy intake in studies where food frequency is recorded, but portion sizes are missing. To enhance accuracy, dietary assessment tools and data bases should incorporate standardised portion sizes. The identified portion sizes can be used to define standard servings in Food Frequency Questionnaires (FFQs) applied in paediatric populations.

Although the sub-analysis stratified by country is informative, the limited sample size in each country and age centile may limit the generalizability of the findings. Children in Spain exhibited food portions greater than other countries for food groups such as fruits, processed fruit products or lean fish, whereas Germany, Belgium, Italy and Poland showed higher portions in specific food groups at certain ages. This trend may reflect cultural differences in feeding practices. However, it is worth taking into account that food portions alone do not reflect actual dietary habits or total intake, as other factors are important, such as daily consumption frequency. For instance, the IDEFICS study reported that the mean daily frequency of fruit and vegetables consumption among children aged 2 to 9 years was higher in Germany (3.13 times/day) compared to Spain (2.50 times/day), Belgium (2.22 times/day) and Italy (2.13 times/day), which could partly explain why fruit and vegetable portions in Germany are smaller [49].

A previous study of our study population reported that Spain and Poland had the highest total daily energy intake, which is consistent with Spain showing larger portions across most food groups. Findings by Jaeger et al., who reported that Spain had the highest consumption of protein and fat among the countries studied align with the larger portions of meat, pulses and fish observed on our study. Conversely, Germany was reported as the country with the lowest daily energy intake, which may relate to the smaller portion sizes observed in our analysis [50]. These findings highlight that portion size and consumption frequency should be jointly considered in dietary assessments, as they influence total energy and nutrients intake.

The similarity in portion sizes between children with a healthy weight at 8 years and the overall sample likely reflects the fact that 29.3% of the total sample had overweight and/or obesity, resulting in only a limited influence on the median portion estimates. Moreover, total energy intake and dietary patterns were not assessed, as these were beyond the scope of this study. The similarity observed between the overall sample and the subsample may also highlight the complex and multifactorial nature of childhood overweight, which has been associated with numerous factors beyond diet, including genetic predisposition, physical activity, and socioeconomic context [51, 52].

### Strengths and limitations

It is important to note that the portion sizes reported in this work include all forms of consumption, whether the food was eaten as a standalone item, incorporated into a recipe, or served as a side dish. One potential limitation of our study is that we captured the amounts consumed of each individual food type, when consumed, but not the total intake per meal combining all foods together. For instance, we recorded separate amounts of vegetables, rice and prawns from a paella, but not the combined weights of the dish. However, our approach, which is very comprehensive, provides other strengths, as it offers a more realistic representation than considering only “regular” portions consumed as main dishes and helps capture the full range of food portions sizes to be consumed a child may encounter within a meal.

Another potential limitation of this study is that feeding practices during the first few months may have somehow evolved since the data were collected. For example, approaches such as “baby-led weaning” have grown in popularity in recent years. In addition, dietary data collected were self-reported, which may have led caregivers to over- or under-report infant dietary intake, potentially influencing the portion sizes recorded. To mitigate this limitation, several strategies to minimize reporting bias were implemented, such us standardising the review process by trained dieticians, assessing the quality of the information collected, correcting inaccurately reported preparations, and identifying misreporting by comparison to the range of estimated energy needs [53–55]. Although these standardization procedures were applied, inter-rater reliability was not formally assessed. Therefore, we cannot rule out the possibility that some differences in nutritional data between sites or evaluators may have influenced the results.

A key strength of our analysis is the use of 3-day dietary records, which provide more detailed, reliable and comprehensive information on food quantities compared to data extracted from Food Frequency Questionnaires (FFQ) or 24-h dietary recalls [56, 57]. Another strength is the expression of food portion intake in percentiles, offering insight into a distribution of typical intake levels across different ages. Nevertheless, although only “poor accuracy” records were excluded, they may have differed systematically from included records—for example, weekends, special-event meals, or days when the child was ill—potentially affecting estimated portion sizes or reducing variability.

## Conclusions

This article presents food portion sizes consumed by infants, toddlers and children across a multicentre European sample highlighting differences between countries as well as across ages and food groups. These findings underscore the need of larger and nationally representative studies are needed to further investigate these patterns and provide more comprehensive understanding of portion size variability. The results presented in this article should be made accessible to researchers, paediatricians, nurses, and dietitian-nutritionists to ensure the consideration of reasonable portion sizes during this stage and to avoid the risks of underfeeding or overfeeding, focusing on respecting hunger and satiety signals from the beginning of life. Future research should also investigate how education programmes considering a wide range of acceptable portion sizes during complementary feeding and childhood relate to later health outcomes.

## Supplementary Information

Below is the link to the electronic supplementary material.

Supplementary file1 (DOCX 32 kb)

Supplementary file2 (DOCX 683 kb)

Supplementary file3 (DOCX 108 kb)

Supplementary file4 (DOCX 524 kb)

Supplementary file5 (XLSX 60 kb)

Supplementary file6 (DOCX 156 kb)

## Data Availability

The datasets generated and/or analysed during the current study are not publicly available due to privacy restrictions concerning individual participants, but are available from the corresponding author on reasonable request.

## Abbreviations

EU CHOP: European Childhood Obesity Project
BMI: Body Mass Index
