# Correction: Characterization of food portion size in children from 6 months to 8 years of age: a descriptive analysis

**DOI:** 10.1007/s00394-026-03965-1

**Published:** 2026-06-15

**Authors:** Irene Hernandez-Perez, Joaquin Escribano, Veit Grote, Berthold Koletzko, Mariona Gispert-Llauradó, Mireia Alcázar, Elvira Verduci, Dariusz Gruszfeld, Louise Etienne, Veronica Luque, CHOP Study Group, H. Demmelmair, U. Handel, I. Pawellek, S. Schiess, S. Verwied-Jorky, M. Weber, R. Closa-Monasterolo, M. Gispert-Llauradó, C. Rubio-Torrents, M. Zaragoza-Jordana, A. Dobrzańska, D. Gruszfeld, R. Janas, A. Wierzbicka, P. Socha, A. Stolarczyk, J. N. Van Hees, J. Hoyos, J. P. Langhendries, F. Martin, P. Poncelet, A. Xhonneux, C. Agostoni, M. Giovannini, S. Scaglioni, F. Vecchi

**Affiliations:** 1 Paediatrics, Nutrition and Development Research Unit, Universitat Rovira I Virgili, Reus, Spain; 2 Institut de Recerca Biomèdica CatSud, Reus, Spain; 3 German Center for Child and Adolescent Health, site Munich, Department of Paediatrics, LMU - Ludwig Maximilians Universität Munich, Dr. Von, Hauner Children’s Hospital, LMU University Hospitals, 80337 Munich, Germany; 4 Metabolic Diseases Unit, Department of Paediatrics, Vittore Buzzi Children’s Hospital, University of Milan, Milan, Italy; 5 Department of Health Sciences, University of Milan, Milan, Italy; 6 Neonatal Department, Children’s Memorial Health Institute, Warsaw, Poland; 7 Groupe Santé CHC, Bd. Patience Et Beaujonc 2, 4000 Liège, Belgium; 8 Serra Hunter Fellow, Universitat Rovira I Virgili, Tarragona, Spain; 9 Children’s University Hospital, University of Munich Medical Center, Munich, Germany; 10 Children’s Memorial Health Institute, Warsaw, Poland; 11 ULB, Brussels, Belgium; 12 CHC St., Vincent, Liège-Rocourt, Belgium; 13 University of Milan, Milan, Italy

**Correction to: European Journal of Nutrition (2026) 65:103** 10.1007/s00394-026-03943-7

In the original version of this article, the authors that part of the CHOP study group were mistakenly listed in the authors’ group.

The correct authors’ group should have been

Irene Hernandez-Perez^1,2^ · Joaquin Escribano^1,2^ · Veit Grote^3^ · Berthold Koletzko^3^ · Mariona Gispert-Llauradó^1,2^ · Mireia Alcázar^1,2^ · Elvira Verduci^4,5^ · Dariusz Gruszfeld^6^ · Louise Etienne^7^ · Veronica Luque^1,3,8^ · CHOP Study Group 

The original article has been corrected.

