# Supplementary material for: Characterization of food portion size in children from 6 months to 8 years of age: a descriptive analysis
Source: Eur J Nutr. 2026 Mar 24;65(3):103. doi: 10.1007/s00394-026-03943-7 (PMC13013196; doi:10.1007/s00394-026-03943-7)
Supplement: Supplementary file 1 — Supplementary file1 (DOCX 32 kb) [file 394_2026_3943_MOESM1_ESM.docx]

Supplementary table 1. Food items included in each food group

| **FOOD GROUPS** | **Clarification/Examples** |
| --- | --- |
| FRUITS | Any type of unprocessed fruit, including berries & wild, malaceus, stone, tropical and citrus fruits |
| PROCESSED FRUITS PRODUCTS | Compotes, fruit concentrated products, convenience food of fruits |
| VEGETABLES | Any type of unprocessed vegetables except the leaf ones |
| LEAFY VEGETABLES | Leafy greens and salads (lettuce, endives, spinach….) |
| SOUPS AND VEGETABLE DISHES | Soups, vegetable dishes |
| READY-TO-EAT INFANT FOODS | Pureed Infant fruits, vegetables, infant meals |
| COW MILK & REGULAR YOGOURT | Milk and curded milk, soya beverage, milk and cheese dishes |
| FLAVORED MILKS | yogourts with added sugars, milk deserts, milk shakes |
| PROCESSED MILK PRODUCTS | ice cream, cream, puddings |
| CHEESE | Hard, soft and cream cheese |
| GRAINS | Whole and refined grains, no-sweetened cereals |
| PROCESSED CEREAL PRODUCTS | “Breakfast” cereals, breadsticks or cereal bars. Convenience food of pasta (filled pasta as tortellini industrially processed and pizza) |
| POTATOES | potatoes (boiled/ steamed/ smashed), tapioca, starchy plant |
| PROCESSED POTATOES | fried/ roasted potatoes, convenience foods of starchy plants |
| PULSES | Lentils, beans, chickpeas |
| NUTS AND SEEDS | Nuts and oil seed products |
| RED MEAT | Beef, veal, pork, lamb, offal |
| WHITE MEAT | Poultry, Horse-, goat-, rabbit meat, winged game |
| PROCESSED MEAT | Animal products, sausages, ham, bacon, cured meat, meat products |
| EGGS | Eggs |
| FATTY FISH | Herrings, mackerel, tuna fish, Salmonidae (salmon, trout), Percoid fish |
| LEAN FISH | Gadoid fish, cod, pollack, red fish, whiting, flatfish, flounder, plaice, sole, Cyprinidae (carp), Crustaceans, conchylia |
| PROCESSED FISH | fish fingers, convenience food of fish, fish products |
| OILS | Olive oil and others |
| OTHER SAUCES (NOT OIL BASED) | Like almond sauce, Bolognese, Neapolitan... |
| SATURATED SPREADS | margarine, butter, vegetable fats |
| ADDED SUGAR | Sugar, honey, sweet spreads, cocoa, jam |
| CAKES, BISCUITS AND PASTRIES | Tarts, cakes, pastries, biscuits, crackers, crepes |
| CONFECTIONARY | Confectionery, sweets, Marzipan, liquorice, croquant, nougat, chocolate, chocolate confectioneries, pralines, sweet products |
| SAVORY SNACKS | Chips, crackers, salty snacks... |
| BEVERAGES | Fruit juices and teas |
| SOFT DRINKS | Caffeinated and soft drinks |
