# Supplementary figures and images for: Characterization of food portion size in children from 6 months to 8 years of age: a descriptive analysis
Source: Eur J Nutr. 2026 Mar 24;65(3):103. doi: 10.1007/s00394-026-03943-7 (PMC13013196; doi:10.1007/s00394-026-03943-7)

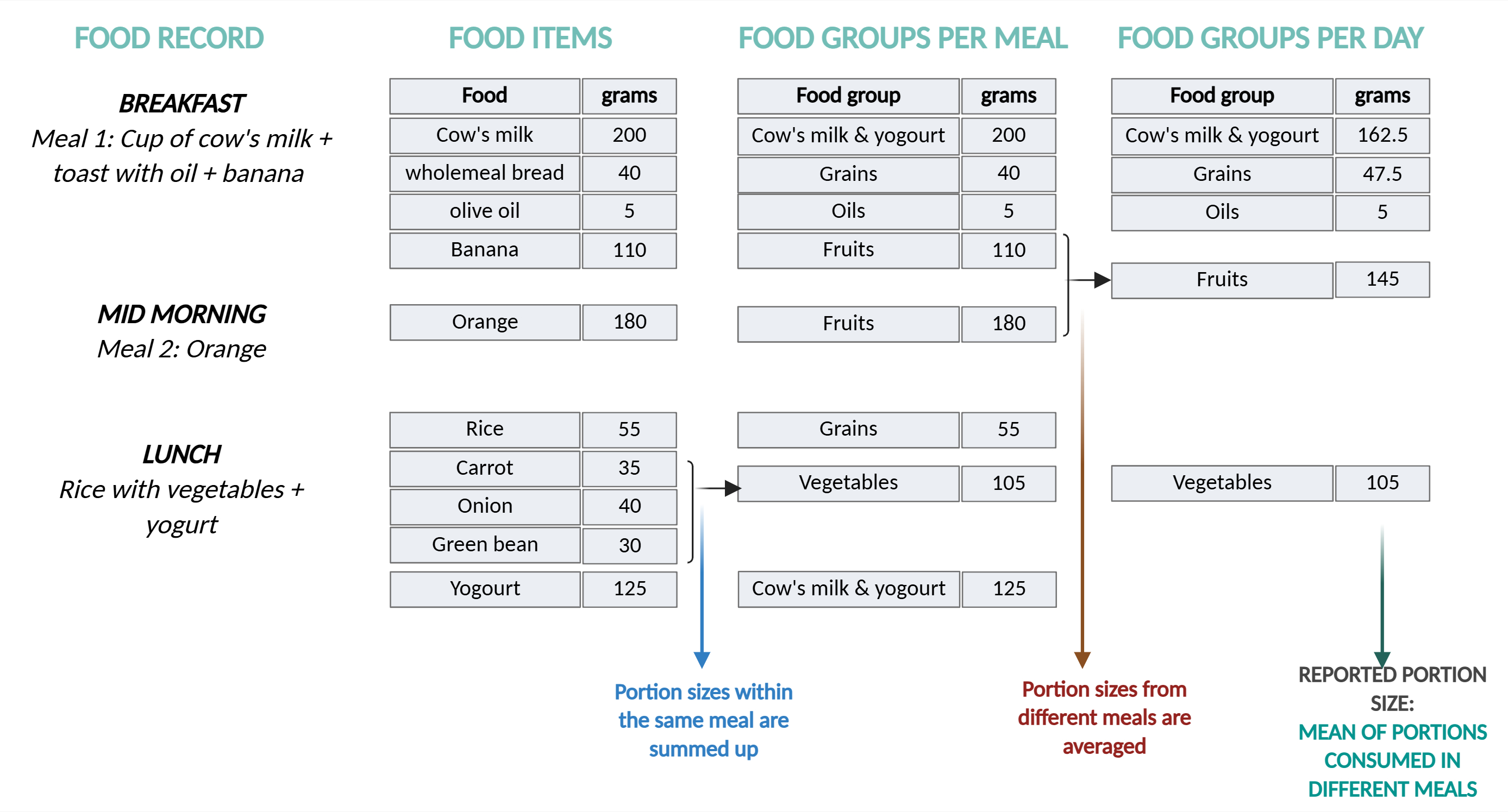

Supplement: Supplementary file 2 — Supplementary file2 (DOCX 683 kb) [file 394_2026_3943_MOESM2_ESM.docx]

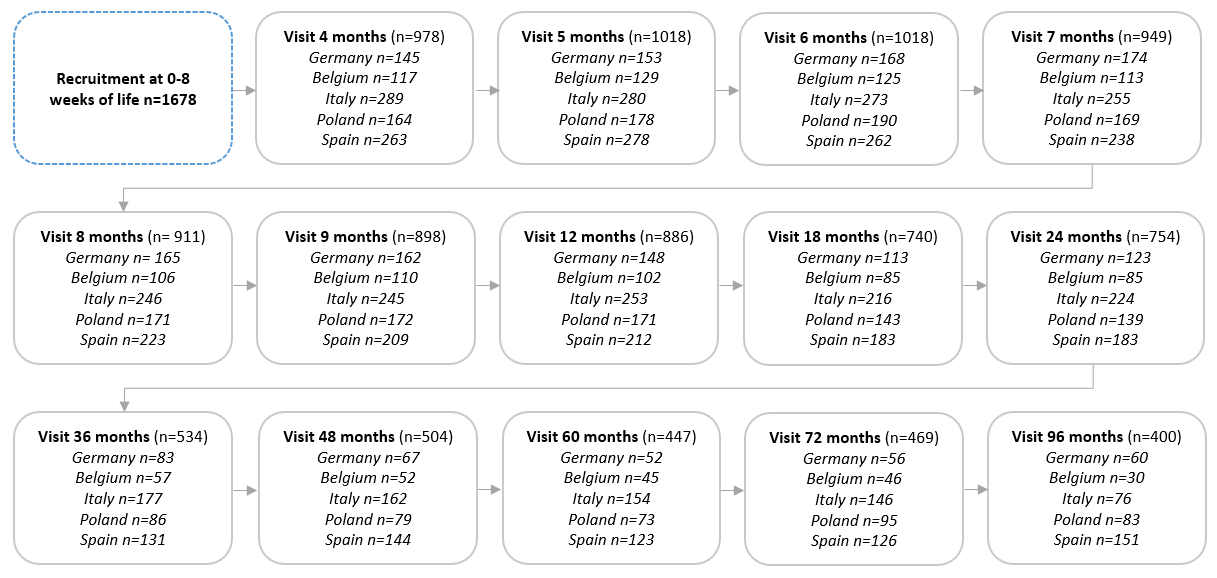

Supplement: Supplementary file 3 — Supplementary file3 (DOCX 108 kb) [file 394_2026_3943_MOESM3_ESM.docx]
