# Supplementary material for: Characterization of food portion size in children from 6 months to 8 years of age: a descriptive analysis
Source: Eur J Nutr. 2026 Mar 24;65(3):103. doi: 10.1007/s00394-026-03943-7 (PMC13013196; doi:10.1007/s00394-026-03943-7)
Supplement: Supplementary file 6 — Supplementary file6 (DOCX 156 kb) [file 394_2026_3943_MOESM6_ESM.docx]

Supplementary Table 4. Food portion size description of foods commonly consumed by normal weight infants at 8 years in percentiles during childhood. n (%) defines the number of children with normal weight at 8 years consuming the food group.

| **Food groups (grams)** | **Age (months)** | | | | | | | | | | | | | | | | | | | | | | | | |
| --- | --- | --- | --- | --- | --- | --- | --- | --- | --- | --- | --- | --- | --- | --- | --- | --- | --- | --- | --- | --- | --- | --- | --- | --- | --- |
|  | 6  n = 368 | | | | | | 7  n = 367 | | | | | | 8  n = 352 | | | | | | 9  n = 358 | | | | | | |
| *Percentile* | *n (%)* | *10* | *25* | *50* | *75* | *90* | *n (%)* | *10* | *25* | *50* | *75* | *90* | *n (%)* | *10* | *25* | *50* | *75* | *90* | *n (%)* | *10* | *25* | *50* | *75* | *90* |  |
| **Fruits** | 168 (45.7) | 43 | 78 | 114 | 159 | 197 | 218 (59.4) | 50 | 75 | 124 | 170 | 207 | 202 (57.4) | 44 | 73 | 112 | 160 | 207 | 221 (61.7) | 50 | 75 | 109 | 155 | 200 |  |
| **Processed fruits products** | 138 (37.5) | 40 | 65 | 98 | 130 | 180 | 190 (51.8) | 43 | 76 | 100 | 130 | 190 | 201 (57.1) | 48 | 71 | 100 | 130 | 200 | 220 (61.5) | 48 | 80 | 112 | 145 | 200 |  |
| **Vegetables** | 137 (37.2) | 9 | 19 | 52 | 87 | 122 | 239 (65.1) | 11 | 30 | 72 | 110 | 136 | 242 (68.8) | 14 | 37 | 72 | 111 | 133 | 262 (73.2) | 11 | 37 | 75 | 105 | 148 |  |
| **Leafy vegetables** | 69 (18.8) | N/R | 2 | 4 | 14 | 50 | 111 (30.2) | 1 | 1 | 2 | 17 | 64 | 119 (33.8) | 1 | 1 | 2 | 20 | 65 | 137 (38.3) | 1 | 2 | 8 | 21 | 63 |  |
| **Soups and vegetable dishes** | 78 (21.2) | 32 | 62 | 108 | 146 | 190 | 82 (22.3) | 37 | 72 | 110 | 160 | 190 | 78 (22.2) | 52 | 76 | 114 | 176 | 190 | 74 (20.7) | 52 | 69 | 95 | 190 | 220 |  |
| **Ready-to-eat infant foods** | 115 (31.3) | 7 | 31 | 76 | 140 | 185 | 177 (48.2) | n/r | 32 | 76 | 138 | 193 | 199 (56.5) | 29 | 55 | 121 | 178 | 210 | 190 (53.1) | 35 | 73 | 130 | 185 | 220 |  |
| **Cow milk and regular yogourt** | 307 (83.4) | 118 | 149 | 178 | 204 | 225 | 309 (84.2) | 113 | 146 | 179 | 206 | 236 | 308 (87.5) | 109 | 144 | 177 | 208 | 237 | 325 (90.8) | 102 | 140 | 176 | 210 | 246 |  |
| **Flavored milks** | 7 (1.9) | 50 | 55 | 75 | 100 | n/r | 22 (6.0) | 38 | 54 | 100 | 114 | 125 | 37 (10.5) | 42 | 55 | 100 | 125 | 130 | 71 (19.8) | 27 | 52 | 97 | 125 | 130 |  |
| **Processed milk products** | 18 (4.9) | 8 | 21 | 40 | 61 | 133 | 54 (14.7) | 8 | 20 | 50 | 74 | 101 | 65 (18.5) | 13 | 25 | 50 | 89 | 125 | 83 (23.2) | 5 | 15 | 50 | 70 | 100 |  |
| **Cheese** | 57 (15.5) | 4 | 4 | 6 | 18 | 50 | 75 (20.4) | 4 | 8 | 14 | 30 | 88 | 87 (24.7) | 4 | 8 | 17 | 42 | 100 | 101 (28.2) | 6 | 10 | 16 | 36 | 79 |  |
| **Grains** | 276 (75.0) | 4 | 8 | 14 | 21 | 46 | 314 (85.6) | 6 | 10 | 15 | 23 | 41 | 321 (91.2) | 7 | 11 | 16 | 26 | 59 | 345 (96.4) | 7 | 12 | 18 | 29 | 57 |  |
| **Potatoes** | 131 (35.6) | 6 | 13 | 42 | 68 | 100 | 229 (62.4) | 7 | 25 | 53 | 80 | 115 | 228 (64.8) | 9 | 26 | 50 | 79 | 108 | 247 (69.0) | 9 | 26 | 53 | 80 | 105 |  |
| **Pulses** | n/r | n/r | n/r | n/r | n/r |  |  |  |  |  |  |  | 4 (1.1) | n/r | 1 | 9 | 48 | n/r | 4 (1.1) | 2 | 13 | 73 | 166 | n/r |  |
| **Red meat** | 13 (3.5) | 5 | 15 | 17 | 36 | 72 | 70 (19.1) | 10 | 18 | 23 | 40 | 50 | 92 (26.1) | 10 | 17 | 29 | 49 | 66 | 131 (36.6) | 12 | 18 | 26 | 48 | 60 |  |
| **White meat** | 33 (9.0) | 13 | 17 | 24 | 40 | 62 | 132 (36.0) | 12 | 20 | 30 | 50 | 76 | 123 (34.9) | 16 | 20 | 33 | 51 | 71 | 148 (41.3) | 12 | 20 | 30 | 48 | 64 |  |
| **Processed meat** | 4 (1.1) | 1 | 1 | 1 | 53 | n/r | 23 (6.3) | 1 | 1 | 7 | 38 | 46 | 29 (8.2) | 1 | 8 | 25 | 33 | 60 | 63 (17.6) | 1 | 4 | 20 | 32 | 50 |  |
| **Eggs** | n/r | n/r | n/r | n/r | n/r | n/r | 22 (6.0) | 3 | 4 | 9 | 17 | 20 | 35 (9.9) | 3 | 7 | 11 | 20 | 33 | 55 (15.4) | 1 | 8 | 17 | 22 | 40 |  |
| **Fatty fish** | n/r | n/r | n/r | n/r | n/r | n/r | n/r | n/r | n/r | n/r | n/r | n/r | n/r | n/r | n/r | n/r | n/r | n/r | 7 (2.0) | 2 | 5 | 30 | 43 | n/r |  |
| **Lean fish and seafood** | n/r | n/r | n/r | n/r | n/r | n/r | 13 (3.5) | 9 | 17 | 25 | 50 | 82 | 22 (6.3) | 12 | 20 | 43 | 50 | 90 | 47 (13.1) | 14 | 26 | 40 | 60 | 94 |  |
| **Oils** | 101 (27.4) | 3 | 4 | 5 | 5 | 9 | 189 (51.5) | 2 | 4 | 5 | 6 | 9 | 185 (52.6) | 2 | 3 | 5 | 6 | 9 | 199 (55.6) | 2 | 4 | 5 | 6 | 9 |  |
| **Other sauces (not oil based)** | n/r | n/r | n/r | n/r | n/r | n/r | n/r | n/r | n/r | n/r | n/r | n/r | 6 (1.7) | 1 | 1 | 1 | 5 | n/r | 15 (4.2) | n/r | n/r | 1 | 3 | 37 |  |
| **Saturated spreads** | 25 (6.8) | n/r | 1 | 2 | 6 | 9 | 56 (15.3) | n/r | 1 | 3 | 5 | 7 | 61 (17.3) | 1 | 1 | 3 | 7 | 9 | 80 (22.3) | n/r | 1 | 3 | 5 | 7 |  |
| **Added sugar** | 28 (8.2) | <1 | 2 | 2 | 4 | 8 | 40 (10.9) | 1 | 2 | 4 | 8 | 11 | 50 (14.2) | 1 | 4 | 6 | 10 | 20 | 63 (18.2) | 1 | 3 | 5 | 10 | 13 |  |
| **Cakes, biscuits and pastries** | 27 (7.3) | 5 | 9 | 15 | 25 | 32 | 53 (14.4) | 5 | 6 | 14 | 20 | 28 | 79 (22.4) | 5 | 6 | 10 | 15 | 59 | 109 (30.4) | 4 | 7 | 12 | 20 | 30 |  |
| **Beverages** | 197 (53.5) | 10 | 24 | 46 | 63 | 96 | 217 (59.1) | 10 | 24 | 40 | 71 | 112 | 204 (58.0) | 9 | 27 | 45 | 72 | 125 | 220 (61.5) | 14 | 30 | 50 | 97 | 158 |  |

n: number of children reporting dietary intake data at each age; n(%)*: number(percentage) of children reporting consumption of the specific food item at each age; consumption of processed cereal products, processed potatoes, processed fish, soft drinks and nuts and seeds were not reported in this age period; N/R Not reported in the specific age or Not Reported by spss due to the small number of observations or low data variability (percentiles coincide with other values or cannot be reliably estimated).

Supplementary Table 4 (Continuation). Food portion size description of foods commonly consumed by normal weight infants at 8 years in percentiles during childhood. n (%) defines the number of children with normal weight at 8 years consuming the food group.

| **Food groups (grams)** | **Age (months)** | | | | | | | | | | | | | | | | | | | | | | | |
| --- | --- | --- | --- | --- | --- | --- | --- | --- | --- | --- | --- | --- | --- | --- | --- | --- | --- | --- | --- | --- | --- | --- | --- | --- |
|  | 12  n = 361 | | | | | | 18  n = 316 | | | | | | 24  n = 328 | | | | | | 36  n = 277 | | | | | |
| *Percentile* | *n (%)* | *10* | *25* | *50* | *75* | *90* | *n (%)* | *10* | *25* | *50* | *75* | *90* | *n (%)* | *10* | *25* | *50* | *75* | *90* | *n (%)* | *10* | *25* | *50* | *75* | *90* |
| **Fruits** | 276 (76.5) | 36 | 60 | 100 | 145 | 215 | 274 (86.7) | 34 | 55 | 85 | 122 | 175 | 281 (85.7) | 37 | 54 | 80 | 108 | 141 | 238 (85.9) | 37 | 63 | 88 | 111 | 130 |
| **Processed fruits products** | 177 (49.0) | 54 | 80 | 100 | 143 | 217 | 82 (25.9) | 80 | 86 | 128 | 190 | 250 | 50 (15.2) | 81 | 100 | 123 | 200 | 250 | 14 (5.1) | 38 | 80 | 127 | 169 | 225 |
| **Vegetables** | 298 (82.5) | 14 | 33 | 62 | 101 | 148 | 300 (94.9) | 16 | 27 | 50 | 80 | 124 | 322 (98.2) | 14 | 27 | 43 | 66 | 98 | 270 (97.5) | 14 | 25 | 41 | 64 | 88 |
| **Leafy vegetables** | 147 (40.7) | 0 | 2 | 8 | 23 | 76 | 166 (52.5) | n/r | 1 | 3 | 15 | 40 | 184 (56.1) | n/r | 1 | 5 | 16 | 48 | 175 (63.2) | n/r | 1 | 4 | 16 | 38 |
| **Soups and vegetable dishes** | 48 (13.3) | 44 | 70 | 107 | 190 | 231 | 18 (5.7) | 15 | 28 | 80 | 193 | 278 | 10 (3.0) | 42 | 75 | 215 | 243 | 295 | 6 (2.2) | 26 | 43 | 140 | 255 | n/r |
| **Ready-to-eat infant foods** | 163 (45.2) | 40 | 78 | 148 | 195 | 250 | 64 (20.3) | 62 | 81 | 170 | 217 | 250 | 41 (12.5) | 62 | 100 | 189 | 230 | 260 | 5 (1.8) | 66 | 136 | 203 | 250 | n/r |
| **Cow milk and regular yogourt** | 339 (93.9) | 100 | 139 | 175 | 214 | 240 | 304 (96.2) | 86 | 134 | 176 | 212 | 246 | 314 (95.7) | 73 | 123 | 176 | 215 | 250 | 262 (94.6) | 69 | 115 | 157 | 200 | 243 |
| **Flavored milks** | 144 (39.9) | 50 | 55 | 91 | 110 | 125 | 194 (61.4) | 37 | 61 | 100 | 125 | 149 | 214 (65.2) | 34 | 56 | 95 | 125 | 150 | 167 (60.3) | 50 | 76 | 107 | 127 | 170 |
| **Milk products processed** | 86 (23.8) | 4 | 10 | 50 | 80 | 125 | 106 (33.5) | 3 | 7 | 11 | 23 | 61 | 124 (37.8) | 4 | 7 | 12 | 32 | 72 | 129 (46.6) | 4 | 10 | 15 | 38 | 65 |
| **Cheese** | 184 (51.0) | 5 | 10 | 17 | 50 | 99 | 244 (77.2) | 5 | 10 | 20 | 47 | 122 | 267 (81.4) | 5 | 10 | 20 | 47 | 100 | 240 (86.6) | 5 | 10 | 19 | 37 | 77 |
| **Grains** | 351 (97.2) | 12 | 17 | 23 | 35 | 55 | 314 (99.4) | 16 | 21 | 28 | 40 | 51 | 326 (99.4) | 17 | 24 | 34 | 44 | 56 | 276 (99.6) | 23 | 29 | 38 | 49 | 59 |
| **Processed cereal products** | 5 (1.4) | 20 | 33 | 50 | 85 | n/r | 9 (2.8) | 12 | 38 | 50 | 70 | n/r | 14 (4.3) | 31 | 40 | 50 | 79 | n/r | 11 (4.0) | 14 | 35 | 70 | 90 | 212 |
| **Potatoes** | 273 (75.6) | 16 | 34 | 65 | 90 | 117 | 261 (82.6) | 21 | 40 | 62 | 91 | 122 | 278 (84.8) | 21 | 37 | 60 | 90 | 135 | 216 (78.0) | 27 | 43 | 60 | 97 | 132 |
| **Processed potatoes** | 4 (1.1) | 5 | 5 | 13 | 121 | n/r | 15 (4.7) | 7 | 30 | 45 | 74 | 131 | 22 (6.7) | 19 | 42 | 60 | 100 | 148 | 26 (9.4) | 12 | 31 | 65 | 100 | 108 |
| **Pulses** | 30 (8.3) | 5 | 10 | 34 | 73 | 125 | 44 (13.9) | 8 | 14 | 32 | 98 | 156 | 52 (15.9) | 5 | 17 | 32 | 120 | 160 | 32 (11.6) | 11 | 29 | 61 | 100 | 191 |
| **Nuts and seeds** | 17 (4.7) | <1 | 1 | 3 | 15 | 45 | 24 (7.6) | n/r | 1 | 3 | 6 | 10 | 40 (12.2) | <1 | 1 | 5 | 8 | 17 | 36 (13.0) | <1 | 2 | 6 | 12 | 22 |
| **Red meat** | 173 (47.9) | 14 | 20 | 35 | 50 | 70 | 221 (69.9) | 11 | 25 | 36 | 52 | 80 | 258 (78.7) | 16 | 25 | 40 | 63 | 95 | 223 (80.5) | 13 | 25 | 39 | 60 | 86 |
| **White meat** | 180 (49.9) | 15 | 24 | 35 | 57 | 80 | 176 (55.7) | 22 | 30 | 46 | 70 | 100 | 184 (56.1) | 20 | 33 | 54 | 83 | 110 | 147 (53.1) | 20 | 39 | 57 | 74 | 106 |
| **Processed meat** | 165 (45.7) | 2 | 13 | 25 | 40 | 57 | 235 (74.4) | 6 | 18 | 30 | 48 | 91 | 263 (80.2) | 11 | 20 | 32 | 50 | 84 | 243 (87.7) | 14 | 20 | 33 | 51 | 85 |
| **Eggs** | 125 (34.6) | 3 | 8 | 19 | 43 | 60 | 202 (63.9) | 4 | 8 | 23 | 50 | 60 | 223 (68.0) | 4 | 10 | 25 | 50 | 60 | 211 (76.2) | 3 | 7 | 22 | 41 | 60 |
| **Fatty fish** | 16 (4.4) | 7 | 21 | 39 | 54 | 93 | 28 (8.9) | 10 | 22 | 35 | 69 | 128 | 375 (114.3) | 5 | 18 | 36 | 60 | 101 | 50 (18.1) | 6 | 15 | 36 | 63 | 100 |
| **Lean fish and seafood** | 101 (28.0) | 20 | 35 | 50 | 76 | 108 | 102 (32.3) | 23 | 36 | 58 | 76 | 117 | 112 (34.1) | 22 | 45 | 76 | 110 | 150 | 94 (33.9) | 19 | 40 | 63 | 100 | 145 |
| **Processed fish** | n/r | n/r | n/r | n/r | n/r | n/r | 18 (5.7) | 23 | 30 | 53 | 72 | 101 | 24 (7.3) | 16 | 30 | 45 | 73 | 115 | 36 (13.0) | 25 | 45 | 60 | 90 | 117 |
| **Oils** | 231 (64.0) | 2 | 4 | 5 | 7 | 10 | 265 (83.9) | 2 | 3 | 5 | 7 | 10 | 284 (86.6) | 2 | 4 | 6 | 8 | 10 | 251 (90.6) | 2 | 4 | 6 | 9 | 11 |
| **Other sauces (not oil based)** | 32 (8.9) | n/r | 1 | 3 | 8 | 12 | 71 (22.5) | n/r | 1 | 4 | 7 | 12 | 98 (29.9) | 1 | 3 | 6 | 11 | 15 | 105 (37.9) | 1 | 3 | 7 | 13 | 20 |
| **Saturated spreads** | 157 (43.5) | 1 | 2 | 4 | 5 | 8 | 223 (70.6) | 1 | 2 | 4 | 5 | 7 | 264 (80.5) | 1 | 2 | 3 | 5 | 8 | 222 (80.1) | 2 | 3 | 5 | 7 | 9 |
| **Added sugar** | 117 (32.4) | 1 | 4 | 6 | 10 | 15 | 201 (63.6) | <1 | 3 | 5 | 9 | 13 | 237 (72.3) | 1 | 4 | 6 | 10 | 14 | 231 (83.4) | 3 | 5 | 8 | 11 | 15 |
| **Cakes, biscuits and pastries** | 183 (50.7) | 6 | 8 | 14 | 25 | 31 | 223 (70.6) | 8 | 11 | 18 | 28 | 40 | 243 (74.1) | 11 | 16 | 23 | 38 | 57 | 234 (84.5) | 14 | 20 | 28 | 36 | 57 |
| **Confectionary** | 20 (5.5) | 2 | 10 | 15 | 21 | 28 | 80 (25.3) | 4 | 6 | 12 | 17 | 28 | 132 (40.2) | 4 | 7 | 11 | 18 | 30 | 167 (60.3) | 4 | 9 | 15 | 22 | 31 |
| **Savory snacks** | 17 (4.7) | 1 | 2 | 5 | 22 | 63 | 42 (13.3) | 1 | 2 | 14 | 31 | 50 | 48 (14.6) | 1 | 4 | 15 | 32 | 80 | 69 (24.9) | 1 | 5 | 25 | 40 | 70 |
| **Beverages** | 205 (56.7) | 11 | 30 | 60 | 98 | 136 | 214 (67.7) | 18 | 47 | 98 | 125 | 200 | 246 (75.0) | 24 | 60 | 107 | 161 | 200 | 221 (79.8) | 39 | 85 | 138 | 200 | 200 |
| **Soft drinks** | n/r | n/r | n/r | n/r | n/r | n/r | n/r | n/r | n/r | n/r | n/r | n/r | 16 (4.9) | 5 | 72 | 100 | 130 | 188 | 36 (13.0) | 30 | 76 | 118 | 200 | 233 |

n: number of children reporting dietary intake data at each age; n(%)*: number(percentage) of children reporting consumption of the specific food item at each age; N/R Not reported in the specific age or Not Reported by spss due to the small number of observations or low data variability (percentiles coincide with other values or cannot be reliably estimated).

Supplementary table 4 (Continuation). Food portion size description of foods commonly consumed by normal weight infants at 8 years in percentiles during childhood. n (%) defines the number of children with normal weight at 8 years consuming the food group.

| **Food groups (grams)** | **Age (months)** | | | | | | | | | | | | | | | | | | | | | | | |
| --- | --- | --- | --- | --- | --- | --- | --- | --- | --- | --- | --- | --- | --- | --- | --- | --- | --- | --- | --- | --- | --- | --- | --- | --- |
|  | 48  n = 278 | | | | | | 60  n = 252 | | | | | | 72  n = 267 | | | | | | 96  n = 278 | | | | | |
| *Percentile* | *n (%)* | *10* | *25* | *50* | *75* | *90* | *n (%)* | *10* | *25* | *50* | *75* | *90* | *n (%)* | *10* | *25* | *50* | *75* | *90* | *n (%)* | *10* | *25* | *50* | *75* | *90* |
| **Fruits** | 249 (89.6) | 46 | 69 | 92 | 119 | 137 | 220 (87.3) | 53 | 78 | 100 | 125 | 150 | 245 (91.8) | 48 | 72 | 100 | 125 | 150 | 242 (87.1) | 50 | 73 | 100 | 125 | 149 |
| **Processed fruits products** | 8 (2.9) | 80 | 93 | 100 | 220 | N/R | 6 (2.4) | 30 | 75 | 90 | 100 | N/R | 9 (3.4) | 90 | 90 | 95 | 114 | N/R | 9 (3.2) | 90 | 90 | 100 | 225 | N/R |
| **Vegetables** | 274 (98.6) | 20 | 28 | 44 | 65 | 88 | 245 (97.2) | 15 | 27 | 41 | 63 | 94 | 258 (96.6) | 22 | 31 | 48 | 67 | 98 | 273 (98.2) | 21 | 36 | 54 | 82 | 116 |
| **Leafy vegetables** | 185 (66.5) | 1 | 1 | 7 | 23 | 54 | 158 (62.7) | 1 | 2 | 9 | 26 | 47 | 182 (68.2) | 1 | 2 | 10 | 27 | 50 | 185 (66.5) | 1 | 2 | 11 | 30 | 59 |
| **Cow milk and regular yogourt** | 267 (96.0) | 81 | 125 | 168 | 200 | 238 | 242 (96.0) | 81 | 120 | 165 | 200 | 247 | 249 (93.3) | 73 | 110 | 156 | 191 | 238 | 269 (96.8) | 69 | 112 | 157 | 200 | 250 |
| **Flavored milks** | 179 (64.4) | 50 | 73 | 110 | 136 | 178 | 168 (66.7) | 50 | 82 | 110 | 137 | 179 | 170 (63.7) | 24 | 78 | 110 | 127 | 185 | 163 (58.6) | 11 | 80 | 124 | 136 | 165 |
| **Milk products processed** | 136 (48.9) | 5 | 10 | 19 | 49 | 80 | 127 (50.4) | 5 | 10 | 24 | 50 | 89 | 154 (57.7) | 6 | 11 | 24 | 50 | 75 | 146 (52.5) | 10 | 15 | 30 | 61 | 89 |
| **Cheese** | 236 (84.9) | 5 | 12 | 19 | 30 | 67 | 222 (88.1) | 5 | 12 | 20 | 38 | 65 | 240 (89.9) | 8 | 15 | 21 | 36 | 76 | 243 (87.4) | 8 | 13 | 22 | 40 | 61 |
| **Grains** | 277 (99.6) | 27 | 32 | 43 | 54 | 70 | 252 (100.0) | 28 | 36 | 46 | 58 | 73 | 267 (100.0) | 29 | 36 | 48 | 66 | 84 | 277 (99.6) | 36 | 45 | 61 | 85 | 118 |
| **Processed cereal products** | 25 (9.0) | 21 | 40 | 60 | 95 | 100 | 15 (6.0) | 30 | 60 | 70 | 100 | 138 | 28 (10.5) | 19 | 53 | 73 | 100 | 123 | 21 (7.6) | 24 | 73 | 100 | 145 | 171 |
| **Potatoes** | 224 (80.6) | 25 | 44 | 65 | 100 | 136 | 199 (78.9) | 28 | 45 | 67 | 93 | 127 | 213 (79.8) | 30 | 49 | 71 | 99 | 142 | 222 (79.9) | 40 | 64 | 92 | 124 | 161 |
| **Processed potaoes** | 22 (7.9) | 38 | 50 | 75 | 100 | 127 | 19 (7.5) | 35 | 50 | 75 | 100 | 128 | 24 (9.0) | 38 | 50 | 64 | 100 | 135 | 23 (8.3) | 24 | 50 | 75 | 100 | n/r |
| **Pulses** | 43 (15.5) | 14 | 31 | 64 | 115 | 176 | 41 (16.3) | 11 | 35 | 70 | 114 | 158 | 40 (15.0) | 13 | 27 | 65 | 145 | 200 | 41 (14.7) | 30 | 44 | 85 | 133 | 200 |
| **Nuts and seeds** | 57 (20.5) | n/r | n/r | 3 | 12 | 20 | 51 (20.2) | n/r | n/r | 3 | 12 | 27 | 52 (19.5) | n/r | 1 | 6 | 19 | 33 | 58 (21.2) | <1 | 2 | 10 | 22 | 39 |
| **Red meat** | 236 (84.9) | 19 | 30 | 46 | 66 | 93 | 222 (88.1) | 19 | 30 | 50 | 75 | 118 | 232 (86.9) | 19 | 31 | 48 | 70 | 100 | 242 (87.1) | 20 | 37 | 59 | 80 | 109 |
| **White meat** | 165 (59.4) | 22 | 38 | 57 | 80 | 106 | 153 (60.7) | 26 | 40 | 60 | 99 | 114 | 175 (65.5) | 29 | 47 | 67 | 90 | 120 | 172 (61.9) | 40 | 60 | 81 | 109 | 140 |
| **Processed meat** | 246 (88.5) | 12 | 20 | 30 | 50 | 71 | 233 (92.5) | 13 | 20 | 34 | 55 | 89 | 238 (89.1) | 14 | 20 | 32 | 50 | 83 | 255 (91.8) | 16 | 23 | 34 | 48 | 75 |
| **Eggs** | 217 (78.1) | 4 | 8 | 19 | 34 | 55 | 202 (80.2) | 4 | 9 | 20 | 38 | 60 | 211 (79.0) | 5 | 10 | 20 | 33 | 60 | 238 (85.6) | 5 | 10 | 25 | 41 | 60 |
| **Fatty fish** | 48 (17.3) | 3 | 10 | 29 | 75 | 103 | 57 (22.6) | 8 | 14 | 26 | 46 | 100 | 58 (21.7) | 10 | 19 | 27 | 51 | 98 | 64 (23.0) | 10 | 20 | 40 | 60 | 100 |
| **Lean fish and seafood** | 100 (36.0) | 21 | 39 | 71 | 105 | 137 | 87 (34.5) | 26 | 42 | 68 | 100 | 130 | 89 (33.3) | 25 | 44 | 61 | 80 | 130 | 93 (33.5) | 30 | 42 | 76 | 105 | 161 |
| **Processed fish** | 39 (14.0) | 2 | 20 | 50 | 80 | 90 | 48 (19.0) | 10 | 35 | 75 | 90 | 120 | 45 (16.9) | 14 | 39 | 60 | 83 | 115 | 45 (16.2) | 4 | 30 | 69 | 100 | 120 |
| **Oils** | 253 (91.0) | 3 | 5 | 7 | 9 | 12 | 230 (91.3) | 3 | 5 | 7 | 10 | 12 | 251 (94.0) | 4 | 5 | 8 | 10 | 13 | 267 (96.0) | 4 | 6 | 9 | 12 | 15 |
| **Other sauces (not oil based)** | 120 (43.2) | 1 | 3 | 6 | 11 | 20 | 96 (38.1) | 2 | 4 | 10 | 16 | 26 | 122 (45.7) | 1 | 4 | 8 | 12 | 20 | 133 (47.8) | 2 | 6 | 10 | 15 | 21 |
| **Saturated spreads** | 212 (76.3) | 2 | 4 | 5 | 7 | 10 | 190 (75.4) | 2 | 4 | 6 | 8 | 11 | 206 (77.2) | 2 | 4 | 6 | 8 | 11 | 214 (77.0) | 3 | 4 | 6 | 9 | 12 |
| **Added sugar** | 243 (87.4) | 4 | 6 | 9 | 13 | 21 | 223 (88.9) | 4 | 5 | 9 | 12 | 19 | 243 (91.0) | 3 | 5 | 10 | 14 | 20 | 255 (91.7) | 4 | 7 | 10 | 14 | 19 |
| **Cakes, biscuits and pastries** | 232 (83.5) | 15 | 22 | 30 | 42 | 59 | 221 (87.7) | 20 | 27 | 36 | 50 | 67 | 234 (87.6) | 18 | 25 | 35 | 50 | 66 | 231 (83.1) | 20 | 29 | 40 | 54 | 78 |
| **Confectionary** | 188 (67.6) | 5 | 10 | 15 | 21 | 27 | 171 (67.9) | 5 | 9 | 15 | 22 | 32 | 177 (66.3) | 7 | 10 | 16 | 25 | 40 | 188 (67.6) | 7 | 10 | 17 | 26 | 42 |
| **Savory snacks** | 90 (32.4) | 1 | 14 | 25 | 36 | 66 | 85 (33.7) | 1 | 2 | 25 | 59 | 116 | 97 (36.3) | 1 | 15 | 30 | 50 | 100 | 89 (32.0) | 1 | 15 | 23 | 40 | 90 |
| **Beverages** | 229 (82.4) | 51 | 101 | 150 | 200 | 200 | 197 (78.2) | 54 | 103 | 151 | 200 | 203 | 209 (78.3) | 27 | 100 | 163 | 200 | 211 | 209 (75.2) | 37 | 108 | 179 | 200 | 240 |
| **Soft drinks** | 46 (16.5) | 50 | 86 | 150 | 200 | 200 | 60 (23.8) | 62 | 102 | 150 | 200 | 264 | 71 (26.6) | 100 | 120 | 200 | 200 | 265 | 100 (36.0) | 100 | 143 | 200 | 230 | 330 |

n: number of children reporting dietary intake data at each age; n(%)*: number(percentage) of children reporting consumption of the specific food item at each age; consumption of soups and vegetable dishes and ready-to-eat infant foods were not reported in this age period; N/R Not reported in the specific age or Not Reported by spss due to the small number of observations or low data variability (percentiles coincide with other values or cannot be reliably estimated).
